# Advancements in the Treatment of Metastatic Breast Cancer (MBC): The Role of Ixabepilone

**DOI:** 10.1155/2012/703858

**Published:** 2012-05-08

**Authors:** Massimo Cristofanilli

**Affiliations:** Department of Medical Oncology, G. Morris Dorrance Jr. Endowed Chair in Medical Oncology, Fox Chase Cancer Center, 333 Cottman Avenue, Room C315, Philadelphia, PA 19111-2497, USA

## Abstract

Successful management of breast cancer in the metastatic setting is often confounded by resistance to chemotherapeutics, in particular anthracyclines and taxanes. The limited number of effective treatment options for patients with more aggressive biological subtypes, such as triple-negative metastatic breast cancer, is especially concerning. As such, a therapy clinically proven to be effective in this subtype would be of great value. Ixabepilone, a novel synthetic lactam analog of epothilone B, demonstrated better clinical outcomes in metastatic disease, particularly in triple-negative breast cancer. Most recently, studies have shown the activity of ixabepilone in the neoadjuvant setting, suggesting a role for this drug in primary disease. Notably, treating in the neoadjuvant setting might allow clinicians to explore the predictive value of biomarkers and response to treatment, as pharmacogenomic approaches to therapy continue to evolve. In this article, we review the efficacy and safety data of ixabepilone as a monotherapy and as a component of combination therapy for metastatic and primary breast cancer.

## 1. Introduction

Breast cancer is the most prevalent malignancy in women and in its metastatic state, the second most common cause of mortality [1]. In 2011, an estimated 230,480 of women were diagnosed with new cases of invasive breast cancer in the United States and 39,520 died from the disease [2]. Despite advances in treatment strategies, metastatic breast cancer (MBC) remains incurable and the goals of therapy range from symptom palliation to extending survival. Treatment selection is based on a series of factors including performance status, site of metastasis, and subtype of disease [3]. Breast tumors can be classified into 5 intrinsic subtypes based on differences in patterns of gene expression: (1) basal-like; (2) human epithelial receptor-2-positive (HER2)/neu, also known as ErbB2+; (3) luminal-like A; (4) luminal-like B; and (5) the normal breast tissue-like subtype [4]. As such, breast cancer treatments can be categorized as: (a) estrogen receptor (ER)-positive and thus, treatable by endocrine therapy; (b) HER2 overexpressed/amplified, treatable with trastuzumab or other HER2-targeted therapies; or (c) triple-negative (TN), with no current specific therapeutic biomarker, typically treated with cytotoxic chemotherapy. This review will outline the treatment strategies in the management of MBC and focus on the role of novel cytotoxic agents, particularly ixabepilone, in this disease.

## 2. The Management of MBC

Breast cancer tumor subtypes are characterized by different clinical outcomes [5]. Basal-like tumors are primarily ER-negative, strongly associated with mutations in the breast cancer susceptibility gene 1 (*BRCA1*), and have a very poor clinical outcome. BRCA1 is a tumor suppressor that functions in many DNA damage response pathways (i.e., homologous recombination and nonhomologous end-joining during DNA double-strand break repair). HER2/neu tumors are typified by overexpression of *ErbB2* and genes involved in the *ras* pathway [6]. Similar to basal-like tumors, they are primarily ER-negative and associated with a poor clinical outcome. Conversely, luminal-like A and B tumors are primarily ER-positive, with luminal-like A providing the best clinical outcome of all subtypes, whereas luminal-like B tumors are associated with an intermediate outcome. Tumors from patients with the normal breast tissue-like subtype tend to have the lowest expression of genes associated with cell proliferation and thus would be expected to have a favorable clinical outcome [7].

 Managing MBC can be extremely challenging, as resistance to standard-of-care chemotherapeutics (i.e., anthracyclines and taxanes) is a major cause of treatment failure [8–10]. Moreover, the widespread use of anthracycline and taxane in the adjuvant setting theoretically contributes to an increase of the proportion of patients with resistance to these drugs for whom other effective agents are necessary for treatment of recurrent disease. This condition is particularly relevant for patients with TN breast cancer, a subset of disease associated with more aggressive features and higher recurrence rate [11]. The most recent investigations explored the various mechanisms of drug resistance and attempted to develop agents that could target those survival pathways. Several preclinical models identified overexpression of neuronal-*β* tubulin III and IV isotopes associated with resistance to taxanes [12, 13]. These observations were confirmed in subsequent clinical studies in patients with MBC treated with first line paclitaxel further confirming the critical role of *β* tubulin in the development of drug resistance [14, 15]. More recently, poly(ADP-ribose) polymerase (PARP) inhibitors were extensively evaluated for their activity and clinical benefit especially for women with TN disease, as PARP inhibitors have shown promising results in mammary tumor models [16, 17].

 Although typically regarded as pro-survival under physiological conditions, studies in which PARP1 was inhibited during S-phase in *BRCA1*- and *BRCA2*-deficient cells have shown that PARP1 inhibition causes genomic instability, cell-cycle arrest, and apoptosis, as these cells were defective in homology-directed double-strand break repair mechanisms [17–19] imparted by unmutated *BRCA1*. Importantly, hereditary breast cancers due to mutations in *BRCA1* are mainly of the basal-like or TN subtypes suggesting the utility of PARP1 inhibitors in treatment of patients with TN breast cancer.

 Early clinical results with the PARP1 inhibitor iniparib (BSI-201) in 123 patients with TN breast cancer were encouraging and showed that the addition of iniparib to treatment with gemcitabine plus carboplatin significantly increased median overall survival (OS, 12.3 versus 7.7 months; *P* = 0.01), median progression-free survival (PFS, 5.9 versus 3.6 months; *P* = 0.01), and objective response rate (ORR, 52% versus 32%; *P* = 0.02) [20]. However, recent results from a randomized phase 3 trial with 519 patients with TN breast cancer did not confirm these data; patients receiving iniparib with gemcitabine and carboplatin experienced no improvement in OS (11.8 versus 11.1 months; *P* = 0.284) and only minor improvement in PFS (5.1 versus 4.1 months; *P* = 0.027) compared with patients receiving chemotherapy alone [21]. This study did not meet the prespecified significance criteria for PFS and OS; ongoing molecular analyses of patients' tumor samples may identify cancer subtypes sensitive to PARP1 inhibition.

 Novel cytotoxic agents would be particularly useful for patients with anthracycline and/or taxane-resistant disease. Eribulin and ixabepilone are microtubule-targeting agents that have recently expanded the armamentarium of available cytotoxic drugs in treatment of MBC.

 Eribulin, a synthetic analog of the marine macrolide halichondrin B, was approved by the US Food and Drug Administration (FDA) and the European Medicines Agency for the treatment of patients with MBC who have received at least 2 prior chemotherapy regimens for late-stage disease. The approval was based on results from a phase 3 trial (EMBRACE) which compared eribulin monotherapy with the physician's choice of treatment in 762 patients with anthracycline- and taxane-pretreated locally recurrent breast cancer or MBC [22]. Patients received 2 to 5 previous chemotherapy regimens, including at least 2 regimens for recurrent or MBC. The physician's choice included any monotherapy, most commonly vinorelbine, gemcitabine, or capecitabine. Eribulin significantly improved median OS compared with the physician's treatment choice (13.1 versus 10.6 months; *P* = 0.041) and also produced a higher response rate (12% versus 5%; *P* = 0.002) but did not show longer PFS (3.7 versus 2.2 months; *P* = 0.137) on independent review. EMBRACE was the first phase 3 study demonstrating a survival benefit for single-agent therapy in heavily-pretreated MBC patients.

 Ixabepilone is a semi-synthetic lactam analog of epothilone B, which belongs to a class of naturally occurring, pro-apoptotic, antineoplastic microtubule-stabilizing compounds [23, 24]. The epothilones, although structurally unrelated to the taxanes, bind to the taxane-binding domain of microtubules. However, the mechanism by which each agent binds to these sites is different [25] and ixabepilone thus maintains activity against paclitaxel-resistant cell lines [26, 27]. Moreover, ixabepilone demonstrated potent activity in a xenograft model with *β*III-tubulin overexpression (Pat-21 breast cancer), and in two xenograft models with an overexpression of drug efflux transporters (Pat-7 ovarian cancer and HCTVM46 colon cancer) [28–30]. 

## 3. Ixabepilone and Its Approved Indications

Among the epothilones, ixabepilone has undergone the most extensive clinical development [31]. Preclinical trials in mouse xenograft models demonstrated that the maximum tolerated dose for ixabepilone was 10 mg/kg [32]. Results from clinical trials of patients treated with ixabepilone 40 mg/m^2^ demonstrated that the exposure was comparable to that of mice treated with ixabepilone 10 mg/m^2^, and that 40 mg/m^2^ offered clinically significant antitumor activity [33]. After determining a clinically effective dose, phase I/II trials in patients with solid tumors investigated several dosing schedules of the Cremophor-based ixabepilone formulation [34]. Subsequent trials described in the next section have identified doses that led to the FDA approval of ixabepilone for the treatment of metastatic or locally advanced breast cancer in patients after failure of an anthracycline and a taxane (in combination with capecitabine) and as a monotherapy after failure of an anthracycline, a taxane, and capecitabine [35].

## 4. Ixabepilone as a Monotherapy or in Combination with Capecitabine: Trials 081, 046, and 048

Three pivotal clinical trials, a phase 2 trial (081) and 2 phase 3 trials (046 and 048), have investigated the efficacy of ixabepilone as a monotherapy and in combination with capecitabine in patients with MBC [36–38].

### 4.1. Trial 081

The single-arm phase 2 trial 081 assessed the efficacy of ixabepilone (40 mg/m^2^ intravenous [IV] over 3 hours every 3 weeks) in women with MBC who were heavily pretreated with an anthracycline, a taxane, and capecitabine. In this trial, patients had significant baseline disease and the majority (88%) had received up to 3 prior therapies in the metastatic setting. Ixabepilone demonstrated ORR among 113 response-assessable patients of 11.5% (95% confidence interval [CI], 6.3%–18.9%) as determined by an independent radiology facility. The investigator-assessed ORR among all 126 patients was 18.3% (95% CI, 11.9%–26.1%). Additional 13% (95% CI, 7.6%–20.9%) of treated patients demonstrated durable (≥6 months) stable disease. Median PFS was 3.1 months (95% CI, 2.7–4.2 months) [36]. The tolerability profile of ixabepilone was acceptable, as treatment-related adverse events were manageable and mostly grade 1 to 2. Selected grade 3 to 4 hematologic, treatment-related adverse events included neutropenia (54%) and fatigue/asthenia (14%), whereas peripheral neuropathy was the most common nonhematologic adverse event. In summary, the results demonstrated that ixabepilone monotherapy has clinically meaningful antitumor activity with a manageable safety profile in patients with MBC resistant to an anthracycline, a taxane, and capecitabine.

### 4.2. Trials 046 and 048

These 2 randomized, open-label phase 3 trials assessed the efficacy of ixabepilone (40 mg/m^2^ IV over 3 hours every 3 weeks) in combination with capecitabine (2000 mg/m^2^ orally on days 1–14 every 3 weeks) versus capecitabine alone (2500 mg/m^2^) on the same schedule. In total, 1973 (*N* = 752  [046] and = 1221  [048]) patients with MBC heavily pretreated and previously exposed to an anthracycline and/or resistant to a taxane, were assessed [37, 38]. The primary endpoint of these studies was PFS.

 In trial 046, PFS associated with ixabepilone in combination with capecitabine was superior to capecitabine as a single agent (hazard ratio [HR] = 0.75; 95% CI, 0.64–0.88; stratified log-rank *P* = 0.0003). Median PFS was prolonged to 5.8 months (95% CI, 5.45–6.97 months) for ixabepilone in combination with capecitabine compared with 4.2 months (95% CI, 3.81–4.50) for capecitabine alone; these data were consistent with investigator-assessed median PFS (5.3 versus 3.8 months; *P* = 0.0011). The ORR was also greater in the combination arm by independent assessment (35% versus 14%; *P* < 0.0001) and by investigator assessment (42% versus 23%) [38]. In trial 048, median PFS (6.2 versus 4.4 months; *P* = 0.0005) for ixabepilone in combination with capecitabine, compared with capecitabine alone, was similar to the median PFS observed in trial 046 [37]. Investigator-assessed ORRs were also consistent with those reported in study 046 (43% versus 29%).

 Ixabepilone plus capecitabine was well tolerated in the phase 3 trials, with a manageable toxicity profile. The most common grade 3/4 adverse event in the combination-therapy arm was peripheral neuropathy, occurring in 23% and 24% of patients in trials 046 and 048, respectively, compared with 0% and 1% of patients in the capecitabine-monotherapy arms, respectively. Fatigue (9–12% versus 3%), myalgia (4–8% versus <1%), and asthenia (6-7% versus 1%) also occurred more frequently in the combination-therapy arm.

## 5. Ixabepilone in First-Line Therapy

### 5.1. Pooled Analysis from Trials 046 and 048

Post-adjuvant breast cancer patients who progress after anthracyclines and taxanes require effective first-line therapies for the treatment of their metastatic disease. This is of particular importance to patients whose tumors demonstrate primary resistance to anthracyclines and taxanes and for those whose anthracycline cumulative exposure is limited by cardiotoxicity. A predefined subgroup analysis of patients with TN breast cancer (*N* = 443)—based on the pooled analysis of trials 046 and 048 (*N* = 1973)—showed that ixabepilone in combination with capecitabine significantly improved PFS (4.2 versus 1.7 months) and increased ORR (31% versus 15%) compared with capecitabine alone [31]. In another analysis from trials 046 and 048, a prespecified subset of post-adjuvant, rapidly relapsing patients (*N* = 293; patients relapsed less than 1 year after completing neoadjuvant/adjuvant therapy) were pooled to assess the efficacy and safety of ixabepilone in combination with capecitabine compared with capecitabine alone. Prespecification of the subset allowed patients to be followed prospectively and because both trials shared similar trial designs and patient populations, the individual subset data were pooled to increase the statistical power [39].

 In patients who received first-line therapy, ixabepilone in combination with capecitabine improved median PFS by 2.8 months compared with capecitabine alone (HR = 0.58; 95% CI, 0.45–0.76; stratified log-rank *P* < 0.0001). Similarly, there was nearly a 2-fold increase in ORR of patients treated with ixabepilone in combination with capecitabine compared with capecitabine alone. Hematologic (i.e., neutropenia and febrile neutropenia) and nonhematologic toxicities (any peripheral neuropathy) associated with the combination were manageable and similar to those observed in the pooled overall population. Compared with capecitabine alone, ixabepilone plus capecitabine was an effective first-line treatment for post-adjuvant, rapidly progressing patients with clinically meaningful improvements in ORR and PFS [39].

### 5.2. Ixabepilone Combinations in MBC

#### 5.2.1. Ixabepilone + Bevacizumab

Preliminary data from preclinical trials showed strong synergistic antitumor activity of ixabepilone in combination with bevacizumab [40]. Recently, a randomized phase 2 trial investigated this combination as first-line therapy for MBC by comparing a weekly ixabepilone-based regimen (16 mg/m^2^ weekly for 3 weeks, every 4 weeks, plus bevacizumab 10 mg/kg every 2 weeks; arm A [*N* = 46]) and an every-3-week ixabepilone-based regimen (40 mg/m^2^ every 3 weeks plus bevacizumab 15 mg/kg every 3 weeks; arm B [*N* = 45]). These experimental treatments were compared with a combination of paclitaxel and bevacizumab (90 mg/m^2^ weekly, plus bevacizumab at the same dose and schedule as arm A; arm C [*N* = 32]) [41]. Final results showed that ORRs were not significantly different among the 3 arms (ORR of 48% [32.9–63.1]; 71% [55.7–83.6]; 63% [43.7–78.9] in arms A, B, C, resp.). Similarly, median PFS also did not show significant differences (9.6 months [6.1–11.7]; 11.9 months [8.7–14.7]; 13.5 months [10.0–18.2] in arms A, B, C, resp.) [41], suggesting that the every-3-week schedules of ixabepilone are as effective as paclitaxel when combined with bevacizumab. The most significant grade 3 or 4 treatment-related nonhematologic and hematologic adverse events included peripheral sensory neuropathy (18%, 24%, 25% in arms A, B, C, resp.) and neutropenia (16%, 60%, 22% in arms A, B, C, resp.); febrile neutropenia rates were ≤2.2% in all arms. Ixabepilone did not appear to exacerbate the toxicity profile of bevacizumab, as the rates of adverse events were similar to those observed with ixabepilone alone at these scheduled doses. Furthermore, the clinical efficacy and tolerability profile demonstrated with ixabepilone in combination with bevacizumab—weekly or every 3 weeks—was comparable to that observed in the phase 3 Eastern Cooperative Oncology Group (ECOG) 2100 trial of bevacizumab in combination with paclitaxel versus paclitaxel alone [42].

 Although the phase 2 trial discussed herein assessed the effect of ixabepilone in combination with bevacizumab, it was the first to compare weekly ixabepilone to the approved every-3-week schedule. Final results from a comparative phase 2 trial of weekly (16 mg/m^2^) or every-3-week ixabepilone (40 mg/m^2^) for 176 patients with MBC suggested that both schedules have an acceptable safety profile [43]. The weekly versus every-3-week schedule produced ORR of 8% (95% CI, 2.5–16.8) versus 14% (95% CI, 6.9–24.1), respectively (*P* = 0.23); median OS of 13.4 months versus 15.0 months, respectively (*P* = 0.21); and median PFS of 2.8 months versus 5.1 months, respectively (*P* = 0.09). These results indicate a trend towards a longer median PFS with every-3-week, compared with weekly ixabepilone. However, the weekly schedule was better tolerated; grade 3/4 adverse events were reported in 28% of patients treated with the weekly schedule and in 69% of patients treated with the every-3-week schedule and included neuropathy (11% versus 20%) and neutropenia (7% versus 40%).

#### 5.2.2. Ixabepilone + Trastuzumab + Carboplatin

Previous studies have shown that the addition of trastuzumab to chemotherapy improves the clinical efficacy profile of patients with MBC whose tumors demonstrate overexpression or amplification of HER2. In one study, the addition of trastuzumab to paclitaxel was associated with a significant improvement in ORR (38% versus 16%, *P* < 0.001) [44, 45]. Likewise, in a phase 3 trial that evaluated the role of carboplatin as a treatment for HER2-positive MBC, the addition of carboplatin to weekly trastuzumab and paclitaxel (175 mg/m^2^ IV every 3 weeks) was associated with a significantly higher ORR (57% versus 36%, *P* = 0.03) and extended PFS (10.7 months versus 7 months, *P* = 0.03) [46, 47].

 Phase 1/2 trials have previously established the efficacy of ixabepilone monotherapy (weekly and every 3 weeks) for patients with MBC [36, 44, 46–51]. As part of combination therapy, phase 1 trials have demonstrated tolerability for ixabepilone in combination with carboplatin [44, 47, 52], and a phase 2 trial of ixabepilone in combination with trastuzumab demonstrated clinically meaningful results in patients with HER2-positive MBC [53]. As such, the ECOG conducted a phase 2 trial (E2103) of weekly ixabepilone in combination with carboplatin and trastuzumab as a first-line treatment for patients with HER2-positive MBC [44]. The primary endpoint of the trial was to determine if an ORR of at least 75% was attainable with the combination. A total of 59 patients were treated with weekly ixabepilone 15 mg/m^2^ IV and carboplatin (area under the curve = 2 IV on days 1, 8, and 15 of a 28-day cycle for a maximum of 6 cycles), plus weekly trastuzumab (4 mg/kg loading dose, then 2 mg/kg IV during chemotherapy, then 6 mg/kg IV every 3 weeks) until disease progression; 39 patients were centrally confirmed to overexpress HER2 (3+ by immunohistochemistry or gene amplification by fluorescent in situ hybridization).

 For all patients treated, the ORR was 44% (95% CI, 31%–58%) and for those patients who had overexpressed or amplified HER2, ORRs were comparable (42%; 95% CI, 26%–58%). Treatment-related toxicities were found to be manageable and low-grade, with grade 3 to 4 neutropenia (49.1%) among the most severe of hematologic toxicities. Notably, there were no reports of febrile neutropenia. Nonhematologic treatment-related adverse events were predominantly mild, with fatigue (11.9%) and sensory neuropathy (6.8%) among the most common grade 3 toxicities. Only 1 patient (1.7%) experienced a grade 4 nonhematologic adverse event of thrombosis/embolism. Although clinically effective as a first-line therapy for HER2-positive MBC, the addition of ixabepilone to carboplatin and weekly trastuzumab did not meet the study primary endpoint. However, the regimen was well tolerated with an efficacy and toxicity profile consistent with that of paclitaxel in combination with carboplatin and trastuzumab. 

## 6. Ixabepilone as a Neoadjuvant Therapy for Breast Cancer

With the availability and access to pretreatment biopsy, the neoadjuvant treatment setting offers the ideal opportunity to identify predictive biomarkers for novel therapeutics and identify single agents or combination regimens likely to impact prognosis. In fact, information regarding biomarkers obtained from tumor biopsies can be assessed and compared with the pathologic complete response in both breast and lymph nodes (pCR) or in the breast only (pCR_B_)—an endpoint strongly associated with disease-free and overall patient survival [54–57]. Based on the encouraging data in MBC, it was deemed appropriate to evaluate the role of ixabepilone in primary disease by using the neoadjuvant model. A phase 2 trial—incorporating an expression analysis of genes identified from a prior clinical investigation as potentially associated with sensitivity/resistance to ixabepilone—evaluated ixabepilone in the neoadjuvant setting [54]. Among such genes was MAP-tau (Tau), a well characterized microtubule stabilizer that is responsible for the bundling, spacing, and assembly of microtubules. MAP-tau may compete for taxane binding sites and/or may be involved in the cooperative binding of paclitaxel to microtubules, therefore potentially having a role in taxanes resistance [58–60]. 

A total of 161 patients with locally advanced breast cancer, defined as primary disease in the breast of ≥3 cm (T2-3), were treated with ixabepilone (40 mg/m^2^ IV over 3 hours every 3 weeks) for 4 or fewer cycles. Of the 161 patients, 42 (26%) had tumors that were TN. Ixabepilone demonstrated antitumor activity comparable with that reported in trials of other single agents in the neoadjuvant setting (pCR_B_= 18% and overall pCR = 17%). Moreover, the highest ORR, 46.1%, was observed in patients with aggressive ER-negative/HER2-positive tumors. These data suggest that ixabepilone may be particular effective in more aggressive cancer subtypes (Table 1). Results also confirmed that ER and *tau *expression are inversely related to pCR_B_ and ixabepilone sensitivity, respectively, confirming what was previously observed in the preclinical setting. The majority of treatment-related toxicities were manageable and mild-to-moderate, with severe adverse events reported in nearly one-third of patients [54]. Grade 3 to 4 neuropathy occurred in ≤3% of patients, and sensory neuropathy was the most common. Grade 3 to 4 neutropenia was observed in 14% of patients and febrile neutropenia was reported in 1% of patients (Table 2). As a neoadjuvant therapy for breast cancer, ixabepilone provided clinically meaningful benefit and had an acceptable safety profile. Moreover, the use of gene expression assays permitted the identification of patients most likely to benefit from neoadjuvant ixabepilone treatment based on expression of the ER and *tau* biomarkers.

## 7. Conclusions

The primary goal of treatment of metastatic disease is optimal palliation. The selection of the most appropriate treatment in this setting requires a careful evaluation of tumor characteristics (disease subtype and location) and patient-related factors (performance status and toxicity profile). In fact, the therapeutic index for various available agents should be considered in the selection of treatment to allow for a more personalized approach. This approach is particularly important to patients with a limited number of therapeutic options, such is the case with patients who have TN MBC. In large prospective clinical trials, ixabepilone has provided evidence of clinical benefit in this subtype and a manageable toxicity profile. Moreover, efficacy in taxane resistance models (*β*-tubulin overexpression) and the recent demonstration of the predictive value of *tau* expression in primary breast cancer tissue, for example, may possibly contribute to further refining the selection of patients who might benefit from ixabepilone as a single agent, or as part of combination therapy in primary and MBC. Future studies should explore the role of this drug in neoadjuvant setting either in combination with biological agents or with other cytotoxic agents to further assess the impact on patients' outcome when used in earlier disease.

## Figures and Tables

**Table 1:** Clinical efficacy of ixabepilone (ixa) as a monotherapy or in combination with a targeted agent and/or chemotherapy in the neoadjuvant and first-line breast cancer settings.

Ixabepilone alone					

Reference	Trial design	Treatment duration	Patients	Dosing schedule	Efficacy
[54]	Phase 2, single arm	Up to 4 cycles	*N* = 161; median age: 55 years; treatment-naive	Per the PI*	pCR in the breast: ER–: 29%ER/PR-negative: 33% ER/PR/HER2-negative: 26%ER–/HER2+: 46.1%%)

[50]	Phase 2, single arm	Until disease progression or unacceptable toxicity for up to 18 cycles	*N* = 93; median age: 52 years; patients received a prior anthracycline-based regimen as adjuvant treatment	Initial dose: Ixa 50 mg/m^2^ as a 1-hour infusion (*n* = 19); Amended dose: Ixa 50 mg/m^2^ as a 3-hour infusion (*n* = 9) Final dose: per the PI* (*n* = 65)	ORR: 41.5% (95% CI, 29.4–54.4%)

Ixabepilone combination therapies					

*Ixabepilone + trastuzumab + carboplatin*					

Reference	Trial design	Treatment duration	Patients	Dosing schedule	Efficacy

[44]	Phase 2, single arm	Up to 6 cycles of chemotherapy; responding patients or those with SD received trastuzumab until PD or study discontinuation	*N* = 59; median age: 51 (all treated patients) *N* = 39; median age: 54 (centrally confirmed HER2+ patients)	Ixa 15 mg/m^2^ and carboplatin AUC = 2 IV on days 1, 8, and 15 every 28 days + weekly trastuzumab (4-mg/kg loading dose, then 2 mg/kg IV) during chemotherapy then 6 mg/kg IV every 3 weeks	All treated patients ORR: 44%Centrally confirmed HER2+ patients ORR: 41%

*Ixabepilone + capecitabine versus capecitabine alone*					

Reference	Trial design	Treatment duration	Patients	Dosing schedule	Efficacy

[39]	Pooled analysis from 2 phase 3, randomized, open-label trials	Until disease progression or unacceptable toxicity	*N* = 149 (ixa + capecitabine); *N* = 144 (capecitabine)	Ixa (per the PI*) + oral capecitabine 2000 mg/m^2^ on day 14 every 3 weeks versus capecitabine 2500 mg/m^2^ alone on day 14 every 3 weeks	ORR: 46% versus 24% PFS: 5.6 versus 2.8 months (HR = 0.58 [95% CI, 0.45–0.76]; *P* < 0.0001)

*Ixabepilone + bevacizumab versus paclitaxel + bevacizumab*					

Reference	Trial design	Treatment duration	Patients	Dosing schedule	Efficacy

[41]	Phase 2, multi-arm	Until disease progression or unacceptable toxicity	[A]: ixa + bevacizumab, *N* = 46; [B]: ixa+bevacizumab, *N* = 45; [C]: paclitaxel + bevacizumab, *N* = 32	[A]: Ixa 16 mg/m^2^ on days 1, 8, and 15 every 28 days + bevacizumab 10 mg/kg IV every 2 weeks; [B] Ixa (per the PI*) + bevacizumab 15 mg/kg IV every 3 weeks; [C] paclitaxel 90 mg/m^2^ IV every 2 weeks + bevacizumab 10 mg/kg IV every 2 weeks	ORR [A]: 48% (95% CI, 32.9–63.1%) [B]: 71% (95% CI, 55.7–83.6%) [C]: 63% (95% CI, 43.7–78.9%) Median PFS (months) [A]: 9.6 (95% CI, 6.1–11.7) [B]: 11.9 (95% CI, 8.7–14.7) [C]: 13.5 (95% CI, 10.0–18.2)

*The recommended dosage of ixabepilone is 40 mg/m^2^ administered intravenously over 3 hours every 3 weeks. Doses for patients with body surface area greater than 2.2 m^2^ should be calculated based on 2.2 m^2^. AUC: area under curve; CI: confidence interval; ER: estrogen receptor; HER2: human epithelial receptor-2-positive; HR: hazard ratio; IV: intravenous; ORR: objective response rate; PD: progressive disease; PFS: progression-free survival; PI: prescribing information; PR: partial response; SD: stable disease.

**Table 2:** Selected Grade 3 or 4 treatment-related adverse events in advanced, first-line, and neoadjuvant breast cancer settings.

Advanced BC					First-line							Neoadjuvant BC
	Ixa alone		Ixa + C	C	Ixa + Carb + T	Ixa alone	Ixa + B	Ixa + B	P + B	Ixa + C	C	Ixa alone

Reference	[36]	[51]	[38]		[44]	[50]	[41]			[39]		[54]

Patient population	A-, T-, C-resistant	T-resistant	A- and T-resistant		HER2+ confirmed by IHC [3+] or FISH	A-resistant	No prior chemo for MBC			No prior chemo for MBC		No prior chemo/radiation

Patient number	126	49	375	377	39	65	46	45	32	149	144	161

Dose	PI*	PI*	Ixa (PI*) + C: 2000 mg/m^2^ on days 1–14 of a 21-day cycle	C: 2500 mg/m^2^ on days 1–14 of a 21-day cycle	[A]	PI*	[B]	[C]	[D]	[E]	[F]	PI*, for 4 or fewer cycles

Grade 3/4 hematologic adverse events (%)												

Febrile neutropenia	3	10	5	<1	0	6	0	2.2	0	5	1	3
Neutropenia	54	53	68	11	49	58	16	60	22	74	9	14

Grade 3/4 nonhematologic adverse events (%)												

PN sensory	14	12^†^	21	0	4	20	18	24	25	23	0	1
PN motor	1	0	5	0	0	5	NP	NP	NP			1

[A] Ixa 15 mg/m^2^ IV and carboplatin AUC = 2 IV on days 1, 8, and 15 every 28 days + weekly trastuzumab (4 mg/kg loading dose, then 2 mg/kg IV) during chemotherapy then 6 mg/kg IV every-3-weeks; [B] Ixa 16 mg/m^2^ IV on days 1, 8, and 15 every 28 days + bevacizumab 10 mg/kg IV every 2 weeks; [C] Ixa (per the PI*) + bevacizumab 15 mg/kg IV every 3 weeks; [D] paclitaxel 90 mg/m^2^ IV every 2 weeks + bevacizumab 10 mg/kg IV every 2 weeks; [E] Ixa (per the PI) + oral capecitabine 1000 mg/m^2^ on day 14 every 3 weeks; [F] capecitabine 1250 mg/m^2^ alone on day 14 every 3 weeks.

*The recommended dosage of ixabepilone is 40 mg/m^2^ administered intravenously over 3 hours every 3 weeks. Doses for patients with body surface area greater than 2.2 m^2^ should be calculated based on 2.2 m^2^.

^†^Sensory neuropathy was assessed and graded according to symptoms as reported by the patient; neurosensory studies were not performed.

B: bevacizumab; BC: breast cancer; C: capecitabine; Carb: carboplatin; FISH: fluorescent in situ hybridization; IHC: immunohistochemistry; Ixa: Ixabepilone; NP: not provided; MBC: metastatic breast cancer; P: paclitaxel; PI: prescribing information; PN: peripheral neuropathy; T: trastuzumab.
